# The conjugative plasmid of a bean-nodulating *Sinorhizobium fredii *strain is assembled from sequences of two *Rhizobium *plasmids and the chromosome of a *Sinorhizobium *strain

**DOI:** 10.1186/1471-2180-11-149

**Published:** 2011-06-25

**Authors:** Laura Cervantes, Patricia Bustos, Lourdes Girard, Rosa Isela Santamaría, Guillermo Dávila, Pablo Vinuesa, David Romero, Susana Brom

**Affiliations:** 1 Programa de Ingeniería Genómica, Centro de Ciencias Genómicas, Universidad Nacional Autónoma de México. Av. Universidad 1001, Cuernavaca, Morelos, CP 62240, México; 2 Programa de Genómica Evolutiva, Centro de Ciencias Genómicas, Universidad Nacional Autónoma de México. Av. Universidad 1001, Cuernavaca, Morelos, CP 62240, México; 3 Programa de Genómica Funcional de Procariotes, Centro de Ciencias Genómicas, Universidad Nacional Autónoma de México. Av. Universidad 1001, Cuernavaca, Morelos, CP 62240, México

## Abstract

**Background:**

Bean-nodulating *Rhizobium etli *originated in Mesoamerica, while soybean-nodulating *Sinorhizobium fredii *evolved in East Asia. *S. fredii *strains, such as GR64, have been isolated from bean nodules in Spain, suggesting the occurrence of conjugative transfer events between introduced and native strains. In *R. etli *CFN42, transfer of the symbiotic plasmid (pRet42d) requires cointegration with the endogenous self-transmissible plasmid pRet42a. Aiming at further understanding the generation of diversity among bean nodulating strains, we analyzed the plasmids of *S. fredii *GR64: pSfr64a and pSfr64b (symbiotic plasmid).

**Results:**

The conjugative transfer of the plasmids of strain GR64 was analyzed. Plasmid pSfr64a was self-transmissible, and required for transfer of the symbiotic plasmid. We sequenced pSfr64a, finding 166 ORFs. pSfr64a showed three large segments of different evolutionary origins; the first one presented 38 ORFs that were highly similar to genes located on the chromosome of *Sinorhizobium *strain NGR234; the second one harbored 51 ORFs with highest similarity to genes from pRet42d, including the replication, but not the symbiosis genes. Accordingly, pSfr64a was incompatible with the *R. etli *CFN42 symbiotic plasmid, but did not contribute to symbiosis. The third segment contained 36 ORFs with highest similarity to genes localized on pRet42a, 20 of them involved in conjugative transfer. Plasmid pRet42a was unable to substitute pSfr64a for induction of pSym transfer, and its own transfer was significantly diminished in GR64 background. The symbiotic plasmid pSfr64b was found to differ from typical *R. etli *symbiotic plasmids.

**Conclusions:**

*S. fredii *GR64 contains a chimeric transmissible plasmid, with segments from two *R. etli *plasmids and a *S. fredii *chromosome, and a symbiotic plasmid different from the one usually found in *R. etli *bv phaseoli. We infer that these plasmids originated through the transfer of a symbiotic-conjugative-plasmid cointegrate from *R. etli *to a *S. fredii *strain, and at least two recombination events among the *R. etli *plasmids and the *S. fredii *genome. As in *R. etli *CFN42, the *S. fredii *GR64 transmissible plasmid is required for the conjugative transfer of the symbiotic plasmid. In spite of the similarity in the conjugation related genes, the transfer process of these plasmids shows a host-specific behaviour.

## Background

Bacterial species belonging to the *Rhizobiaceae *are common inhabitants of the soil and the rhizosphere. Most of them are able to establish a symbiotic relationship with the roots of leguminous plants through the formation of nodules, where bacteria differentiate into nitrogen fixing bacteroids [1]. The genomes of these bacteria contain a circular chromosome. Some, like *Agrobacterium tumefaciens*, also contain a linear chromosome, in addition to a variable number of plasmids, which may carry up to 50% of the genomic sequence. The bacterial genetic information required for the establishment of the symbiosis is usually localized on large plasmids, or in genomic islands [2].

Conjugative transfer is thought to be the most relevant mechanism that contributes to the dissemination and diversification of genetic information, particularly that localized on plasmids. Conjugation systems are constituted by a DNA transfer and replication (Dtr) component, encoded by *tra *genes and a *cis*-acting *oriT *site, and a mating pair formation (Mpf) component, encoded by *trb *genes [3]. Information on the conjugative transfer mechanisms of rhizobial plasmids is still scarce. However, two groups of plasmids containing a functional set of conjugative transfer genes have been described: Type I, which are regulated by quorum-sensing [3-5], and Type II, which have permanently RctA-repressed transfer genes [3,6]. A third type of conjugative plasmids has been recently proposed, represented by the largest plasmids of *R. leguminosarum *bv viciae strains [3]. Some plasmids are mobilizable in the presence of transmissible plasmids, either by cointegration (conduction) [7], or by classical (trans) helper mechanisms [8,9]. Specifically in the bean nodulating type strain *Rhizobium etli *CFN42, we have previously shown that it contains a quorum-sensing regulated self-transmissible plasmid (pRet42a) [5], and that transfer of the symbiotic plasmid (pRet42d) occurs only in the presence of pRet42a. The event requires cointegration of both replicons. This may be achieved through IntA-dependent site-specific recombination between *attA *and *attD *sites, or through RecA-dependent homologous recombination among large sequence segments shared between the replicons. The cointegrate is able to transfer, using the pRet42a-encoded machinery. In the transconjugants, the cointegrate is usually resolved to regenerate the wild-type plasmids, but in a few cases, resolution of the cointegrate leads to the formation of recombinant plasmids that contain segments of each plasmid, pRet42a and pRet42d [7]. Mesoamerica has been identified as the place of origin of bean plants and *Rhizobium etli *bacteria [10], while soybean and its nodulating bacteria (*Sinorhizobium fredii*) originated in East Asia [11]. In the early XVI^th ^century, common beans and their symbionts were transported to Europe and other parts of the world. A survey of bean-nodulating strains in Granada, Spain, showed the presence of strains belonging to five different species: *R. etli*, *R. gallicum*, *R. giardinii*, *R. leguminosarum *and *S. fredii *[12]. The usual host of *Sinorhizobium fredii *strains is soybean (*Glycine soja*), not common bean (*Phaseolus vulgaris*). Nevertheless, the bean-nodulating strains classified as *S. fredii*, were unable to nodulate cvs. Williams or Peking of *Glycine max*. Hybridization of digested genomic DNA with *nodB *and *nifH *genes from *R. etli*, showed a very weak signal [12]. *R. etli *bv phaseoli symbiotic plasmids (pSyms) are characterized by the presence of three copies of *nifH*. The bean-nodulating *S. fredii *strains showed only one copy of this gene [12]. While conjugative transfer may explain the acquisition of new symbiotic features by strains belonging to diverse species, the relationship between *R. etli *and bean-nodulating *S. fredii *is not so easily established. In order to gain further insight into the mechanisms and pathways leading to the generation of new rhizobial strains, in this work we present the analysis of the bean-nodulating *S. fredii *strain GR64, isolated from the soil in Granada. The results indicate that the plasmids present in GR64 likely derived from conjugative transfer and rearrangement events among sequences localized in at least three different replicons, including two different plasmids and a chromosome.

## Results

### Plasmid pSfr64b is required for symbiosis but pSfr64a is dispensable

**Figure 1:**
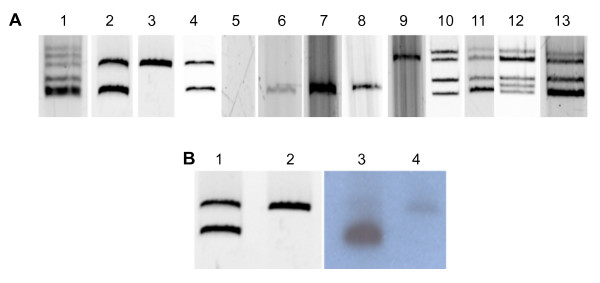
**Eckhardt type gel showing the plasmid profile of *S. fredii *strain GR64 and derivatives, in comparison to *R. etli *CFN42**. Panel A. Ethidium bromide stained Eckhardt gel. Lane 1: CFN42, lane 2: wild type GR64, lane 3: GR64-2, lane 4: GR64-3, lane 5: GR64-4, lane 6: GR64-5, lane 7: GR64-6, lane 8: GMI9023/pSfr64a, lane 9: GMI9023/pSfr64b, lane 10: CFN2001, lane 11: CFN2001-1, lane 12: CFN2001- 2, lane 13: CFN2001-3. Panel B. Ethidium bromide stained Eckhardt gel (lanes 1 and 2), and Southern blot of the plasmid profiles probed with pSfr64a (lanes 3 and 4). Lanes 1 and 3: GR64-1 (GR64/pSfr64a::Tn*5*-GDYN, pSfr64b::Tn*5mob*), lanes 2 and 4: GR64-2 (pSfr64a^-^, pSfr64b::Tn*5mob*).

Strain GR64 contains two plasmids: pSfr64a (183 kb) and pSfr64b (~400 kb) (Figure 1A, Table 1). A band corresponding to a megaplasmid (~1300 kb), has been visualized [13], but is not always clearly apparent in the gels. Plasmid pSfr64b was identified as the symbiotic plasmid [13], because it hybridizes with the *nifH *gene. Nodulation assays confirmed that the genetic information in pSfr64b is necessary and sufficient to establish symbiosis. Table 2 shows that all derivatives carrying pSfr64b, were able to form nodules (GR64, CFN2001-1, GMI9023/pSfr64b), and that the construct lacking pSfr64b (GR64-4) was unable to nodulate beans. Consistent with previous findings [14,15], the number of nodules was decreased in an *Agrobacterium *genomic background. On the other hand, lack of pSfr64a had no effect on the symbiotic process (GR64-2), and its presence in *Agrobacterium *did not confer nodulation capacity to the receptor, indicating that pSfr64a encodes none of the essential symbiotic genes.

**Table 1:** Strains and plasmids used in this study

Strain	Relevant characteristic	Source
***Rhizobium***		
CFN42	wild type *R. etli *(pRet42a to pRet42f)	[58]
CFN2001	CFN42 lacking pRet42a and pRet42d	[37]
CFNX195	CFN42 derivative cured of pRet42a, pRet42d::Tn*5mob*	[32]
GR64	wild type bean-nodulating *S. fredii *(pSfr64a, pSfr64b)	[12]
GR64-1	GR64/pSfr64a::Tn*5*-GDYN, pSfr64b::Tn*5mob*	This work
GR64-2	GR64 cured of pSfr64a, pSfr64b::Tn*5mob*	This work
GR64-3	GR64-2 with pRet42a::Tn*5*-GDYN	This work
GR64-4	GR64 cured of pSfr64a and pSfr64b, Rif^R^	This work
GR64-5	GR64-4/pRet42a::Tn*5*-GDYN	This work
GR64-6	GR64-4/pSfr64a::Tn*5*-GDYN	This work
CFN2001-1	CFN2001/pSfr64b::Tn*5mob*	This work
CFN2001-2	CFN2001/pSfr64b::Tn*5mob*, pRet42a::Tn*5*-GDYN	This work
CFN2001-3	CFN2001/pSfr64b::Tn*5mob*, pSfr64a::Tn*5*-GDYN	This work
***Escherichia coli***		
DH5a	Receptor for transformation	[59]
S17-1	C600::RP-4-2 (Tc::Mu)(Km::Tn7)	[60]
S17/pDR21	Source of Tn*5*-GDYN	[17]
***Agrobacterium tumefaciens***		
GMI9023	C-58 cured of its native plasmids	[35]
UIA143	*recA *pTi^- ^derivative of C58	[61]
GMI9023/pSfr64a	GMI9023 with pSfr64a::Tn*5*-GDYN	This work
GMI9023/pSfr64b	GMI9023 with pSfr64b::Tn*5mob*	This work
**Plasmids**		
pSUP5011	Tn*5mob*	[60]
pRK2013	Conjugation helper	[36]

**Table 2:** Nodulation assay of bean-nodulating strains.*^a^*

Strain	Relevant features	N^o ^nodules/plant*^b^*
CFN42	wild type *R. etli*	57.3 (31.0)
GR64	wild type bean-nodulating *S. fredii*	30.6 (5.3)
CFN2001-1	CFN2001/pSfr64b::Tn*5mob*	31.6 (13.1)
GR64-2	pSfr64a**^-^**, pSfr64b::Tn*5mob*	24 (7.4)
GR64-4	pSfr64a**^-^**, pSfr64b**^-^**	0
GMI9023/pSfr64b	GMI9023 with pSfr64b::Tn*5mob*	4.6 (3.2)
GMI9023/pSfr64a	GMI9023 with pSfr64a::Tn*5*-GDYN	0
GMI9023	wild type	0

*^a ^*Average of three plants

*^b ^*Standard deviation

### Plasmid pSfr64a shares sequences with the *R. etli *pSym, pRet42a, and with the chromosome of *Sinorhizobium fredii *NGR234

**Figure 2:**
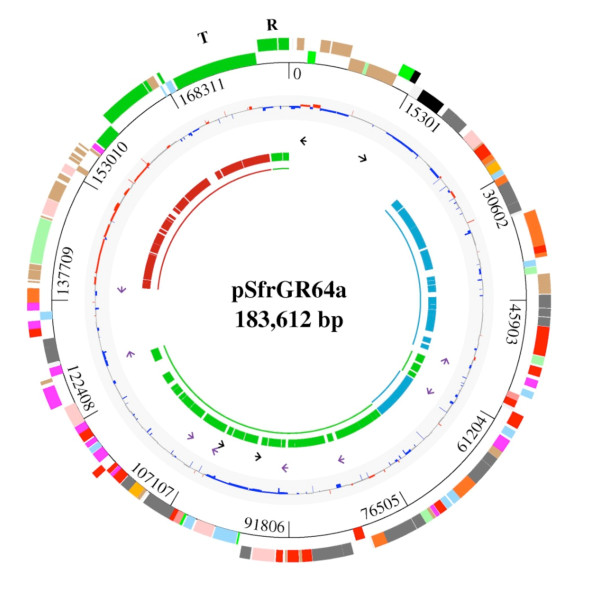
**Structure of plasmid pSfr64a**. Descriptions are presented from the innermost circle outward: regions with homology to pRet42a (red), pRet42d (green) and the chromosome of NGR234 (blue); ORFs with homology to pRet42a (red), pRet42d (green) and the chromosome of NGR234 (blue); transposon-related ORFs: pSFR64a_00003, pSFR64a_00009, pSFR64a_00084, pSFR64a_00088 (black arrows); transposon-related ORFs on pRet42a (PA00138) and pRet42d (PD00033, PD00041, PD00093, PD00124, PD00101, PD00123, PD00041) located nearby to the ORFs where similarity is interrupted (purple arrows); GC content (blue, low GC; gray, medium GC; red, high GC); predicted ORFs on the forward and reverse strands in color code (the colors are according to their functional category as follows: orange, amino acid biosynthesis; light red, biosynthesis of cofactors; pale green, macromolecule biosynthesis; mild red, central intermediary metabolism; red, energy transfer; magenta, degradation; pink, structural elements or cell processes; dark gray, transport; bright green, transposon-related functions; sky blue, transcriptional regulators; green, transfer functions or replication functions; brown, hypotheticals; bone, orphans; black, function not determined). The locations of the replication genes (R) and of the transfer region (T) are indicated.

We sequenced plasmid pSfr64a (GenBank accession number: CP002245). The main features of this plasmid are shown in Figure 2 and Additional File 1. Plasmid pSfr64a is 183 612 bp long. The genetic organization of this plasmid clearly reveals its chimeric nature, since 38 (23%) of the 166 ORFs encoded in the plasmid presented highest similarity to sequences of the chromosome of *Sinorhizobium fredii *NGR234, while 87 (52%) were most similar to ORFs encoded in *R. etli *CFN42 plasmids pRet42a (36 ORFs, 22%) and pRet42d (51 ORFs, 31%).

The functional assignment of the 166 ORFs (Figure 2, Table 3) shows that the plasmid is largely involved in metabolic, transport and conjugative functions.

**Table 3:** Functional assignment of pSfr64a ORFs.

Funtion	N^o ^of ORFs			
	**Total^a^**	**p42d^b^**	**p42a^c^**	**NGR234^d^**

Small Molecule Metabolism	48	25	0	15
Macromolecule Metabolism	5	0	2	1
Chemotaxis	4	2	0	1
Transport of small molecules	28	13	0	11
Transposon - related	4	0	0	0
Replication	3	3	0	0
Conjugation	20	0	20	0
Transcriptional regulation	14	5	0	4
Conserved hypothetical	38	3	14	6
Unknown	2			
Total	166	51	36	38

*^a ^*Number of pSfr64a ORFs assigned to each functional category.

*^b ^*Number of pSfr64a ORFs in each category, with highest similarity to ORFs from pRet42d.

*^c ^*Number of pSfr64a ORFs in each category, with highest similarity to ORFs from pRet42a.

*^d ^*Number of pSfr64a ORFs in each category, with highest similarity to ORFs from the chromosome of NGR234.

**Figure 3:**
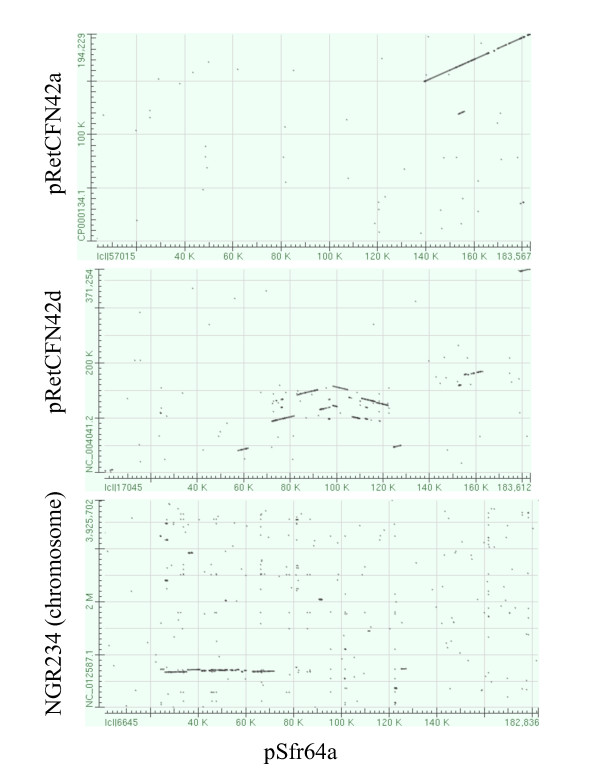
**Colinearity between pSfr64a and other replicons**. Dot matrix view of BLASTN comparisons of pSfr64a vs pRet42a, pRet42d and the chromosome of NGR234.

Among the ORFs shared between pSfr64a and pRet42a, the self-transmissible plasmid of CFN42, most are related to conjugative transfer (20 ORFs), only two were ascribed to macromolecular metabolism. Interestingly, both are related to DNA metabolism, one was classified as a putative nuclease, and the other as a probable DNA methylase. In Figure 3, it can be appreciated that the genomic region shared between pRet42a and pSfr64a is markedly colinear. Colinearity is disrupted by the absence of an homolog to the regulatory gene *cinR *of pRet42a, and the presence of pSfr64a ORFs 147 and 148, which encode hypothetical proteins. The correspondence between pSfr64a and pRetCFN42 ORFs is presented in Additional File 1. Figure 2 shows that the segment of pSfr64a shared with pRet42a has a high GC content, compared to the rest of the plasmid. This feature is also present in the similar pRet42a sequence.

The ORFs similar to the pSym of CFN42 (pRet42d) include the *repABC *genes (Figure 2, Table 3). This is congruent with our finding that pSfr64a and pRet42d are incompatible (data not shown). The pSfr64a-pRet42d-shared ORFs are mainly involved in small molecule metabolism (26 ORFs), and carbohydrate transport (13 ORFs). It is noteworthy that, in spite of the fact that pRet42d carries genes engaged in symbiotic functions, none of these are present in pSfr64a.

Within the region similar to pRet42d (ORFs 46 to 110), the colinearity is restricted to small segments, some of them in inverse orientation. (Figure 3, Additional file 1). The *repABC *genes (pSfr64a ORFs 164 to 166) were adjacent to the transfer region, separated from the other pRet42d genes. It has been amply documented that plasmid pRet42d is subject to frequent genomic rearrangements, due to the presence of reiterations and a high density of insertion sequences [16-20]. *R. etli *ORFs encoding transposon-related proteins located near to the sites where colinearity is disrupted are indicated in Figure 2 (purple arrows) and Additional File 1. For example, pSfr64a ORFs 122 to 146 are colinear with pRet42a ORFs 139 to 162. The adjacent ORF on pRet42a (ORF 138) encodes a transposon-related protein. It is possible that these sequences are related to the generation of rearrangements, causing the interruptions in colinearity. ORFs 114, 115, 116, 117, 118 and 121 show homology to ORFs encoded in another *Rhizobium etli *strain; IE4771 [21]. The genome of this strain has been sequenced, but not assembled, so we cannot assign them to the pSym, although that is their most probable localization.

The majority of the ORFs shared between pSfr64a and the chromosome of NGR234 are related to small molecule metabolism (15 ORFs), and to the transport of small molecules (11 ORFs). As shown in Figure 3 and Additional File 1 this region is also highly colinear with the corresponding genes on the chromosome of NGR234. Data presented in this section suggest that pSfr64a was assembled during evolution as a chimeric structure, harboring segments from two separate *R. etli *plasmids and the chromosome of a *Sinorhizobium *strain, such as NGR234.

### Plasmid pSfr64a is transmissible and required for transfer of pSfr64b

The structural conservation on pSfr64a of genes involved in conjugation, raised the possibility of self-transmissibility of this replicon; therefore, the conjugative capacity of GR64 plasmids was studied. The results (Table 4) show that plasmid pSfr64a is transmissible at a high frequency. The symbiotic plasmid pSfr64b was also able to perform conjugative transfer, but only when pSfr64a was present. We conclude that pSfr64a provides transfer functions to pSfr64b. The process could be similar to what we described for CFN42, where pRet42a induces pSym transfer by cointegration. Alternatively, pSfr64b mobilization could be induced *in trans*. Interestingly, the transfer frequency of this pSym was found to be two orders of magnitude higher than that of *R. etli *CFN42 pSym.

**Table 4:** Transfer frequency of self-transmissible and symbiotic plasmids *^a^*

Donor	Relevant genotype	Transfer Frequency^b^	
		**STP^c^**	**pSym**

CFN42	wild type *R. etli*	10^-2^	10^-6^
CFNX195	CFN42 derivative: pRet42a^-^, pRet42d::Tn*5mob*	-^d^	ND^e^
GR64	wild type *S. fredii*	10^-1^	10^-4^
GR64-2	GR64/pSfr64a**^-^**, pSfr64b::Tn*5mob*	-	ND
GR64-3	GR64-2/pRet42a::Tn*5*-GDYN	ND	ND
GR64-5	GR64/pSfr64a**^-^**, pSfr64b^-^, pRet42a::Tn*5*-GDYN	ND	-
GR64-6	GR64/pSfr64a^-^, pSfr64b^-^, pSfr64a::Tn*5*-GDYN	10^-1^	-
CFN2001-1	CFN2001/pSfr64b::Tn*5mob*	-	ND
CFN2001-2	CFN2001-1/pRet42a::Tn*5*-GDYN	10^-4^	10^-6^
CFN2001-3	CFN2001-1/pSfr64a::Tn*5*-GDYN	ND	ND

*^a ^*Strain GMI9023 was used as receptor. All crosses were repeated at least three times.

*^b ^*Expressed as the number of transconjugants per donor.

*^c ^*STP: Self Transmissible Plasmid

*^d ^*Not done

*^e ^*not detected (transfer frequency <10^-9^).

### Genomic background determines functionality of conjugative plasmids

In order to assess the specificity of pSym transfer induction, we constructed derivatives containing diverse plasmid combinations, in either *R. etli *or *S. fredii *genomic backgrounds, as described in Materials and Methods, and determined the transfer frequency of the self-transmissible and symbiotic plasmids (Table 4). Analysis of a derivative containing the *R. etli *self-transmissible plasmid pRet42a in *S. fredii *background (GR64-3) showed a dramatic decrease in the transfer ability of the plasmid as well as no transfer of the GR64 pSym. These results suggest that the genome of GR64 contains an inhibitor of pRet42a transfer. The decrease in pRet42a transfer could mask its function as helper for pSym transfer induction. The fact that pRet42a transfer is also decreased in a derivative lacking the pSym of GR64 (GR64-5), points to a chromosomal location of the putative inhibitor locus. Similarly, *S. fredii *pSfr64a was unable to perform conjugative transfer or induce transfer of pSfr64b in *R. etli *genomic background (CFN2001-3). Only *R. etli *pRet42a was still able to induce pSfr64b transfer in the *R. etli *background (CFN2001-2).

### The pSym of GR64 differs from the typical *R. etli *pSym

**Figure 4:**
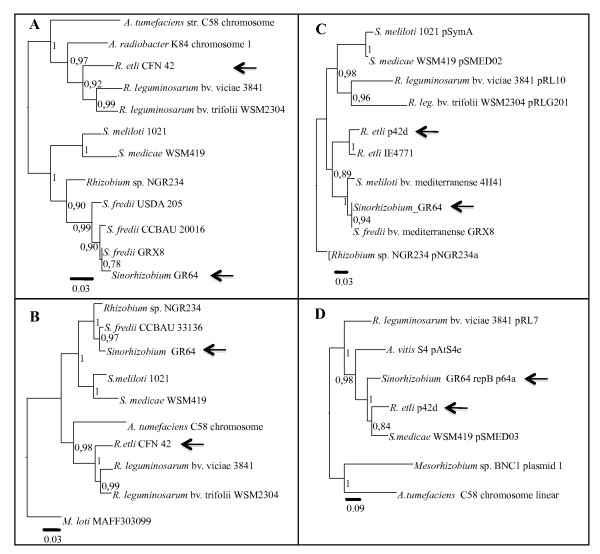
**Phylogeny of *S. fredii *GR64**. Maximum likelihood phylogenetic trees based on chromosomal: (A) *recA*, (B) *rpoB*, and plasmid: (C) *nifH *and (D) *repB *gene fragments. Arrows indicate the localization of *S. fredii *GR64, and *R.etli *CFN42.

To further analyze the bean-nodulating *S. fredii *strain GR64, we performed a phylogenetic analysis with chromosomal genes (*recA, rpoB*), and with the plasmid-encoded genes *nifH *and *repB*. The results (Figure 4) show that, based on the phylogeny of the chromosomal genes, GR64 clusters within the *fredii *clade, while *nifH *and *repB *genes group strain GR64 with other bean-nodulating *Sinorhizobium *strains isolated from the South of Spain (Granada and Sevilla) [22,23] and from the North of Africa (Tunisia) [24] (Figure 4C). The data obtained indicate that GR64 has a *S. fredii *chromosome but carries a pSym that allows nodulation of *Phaseolus*. However, this plasmid differs from typical *R. etli *pSyms in its replication genes, allowing it to coexist with plasmid pSfr64a, which does share its replication genes with the *R. etli *pSym. Another feature that differentiates this pSym is the presence of a single copy of the *nifH *gene.

## Discussion

Genomic comparisons of *S. meliloti*, *A. tumefaciens*, and *R. etli *[25], and between *Rhizobium leguminosarum *bv *viciae *and *Rhizobium etli *[26], have shown that chromosomes are well conserved both in gene content and gene order, whereas plasmids presented few common regions and lacked synteny, except for some pairs of plasmids whose features indicate that they were part of the ancestral genome, and may be considered as secondary chromosomes [26,27]. In *R. etli*, the symbiotic and self-transmissible plasmids are the less conserved replicons [25] with fewer collinear blocks [26].

In this paper we show that a conjugative plasmid from a bean nodulating *S. fredii *strain is formed by large segments of replicons found in strains belonging to different species from diverse geographic origins. These replicons include two plasmids of *R. etli*, and a *S. fredii *chromosome. In GR64, bean-nodulation is provided by pSfr64b. Although the phylogenetic relationship of the GR64 *nifH *gene shows that it is closely related to the *R. etli *gene (Figure 4), pSfr64b differs from the typical *R. etli *pSym in other features (see above).

We have previously reported that *R. etli *pRet42a is able to form a cointegrate with the pSym, and thus promote its conjugative transfer, and that in some cases (10% of the events), resolution of the cointegrate leads to the generation of recombinant plasmids containing segments of both pRet42a and the pSym [7]. Also, the occurrence of frequent genomic rearrangements in rhizobial species has been amply documented [19,20,25,28].

Integrating these data, we propose that the *R. etli *plasmids were transferred to a *S. fredii *strain and recombination events among the plasmids, the chromosome, and possibly another endogenous *S. fredii *plasmid, led to the generation of plasmids pSfr64a and pSfr64b. This would indicate that pSfr64a is an evolutionary "new" plasmid of chimeric origin, that was generated after *R. etli *strains arrived to Europe, following the discovery of America, when bean seeds coated with bacteria were most likely introduced to that continent [29]. It is noteworthy that pSfr64a, in spite of carrying a large segment of chromosomal origin, would not be considered as a secondary chromosome, as it can be cured without affecting the saprophytic phenotype of the strain (data not shown). It is possible that such a plasmid is an "intermediate" in the formation of secondary chromosomes. Other plasmids with a structure similar to that of pSfr64a, have yet to be described. The finding of such a plasmid in a natural environment may be a living example of a pathway that allows shuffling of the *repABC *genes, which has been proposed as a strategy to explain the plasmid diversity of Rhizobium [26]. Also, the fact that the *repABC *genes are located adjacent to the transfer region that is similar to that of pRet42a, and separate from the other sequences that are similar to the *R. etli *pSym, highlights the impact of evolutionary forces leading to this arrangement, which is highly conserved in many plasmids, and must have evolved in a relatively short time period.

Strain NGR234 was isolated in 1965 by M. J. Trinick, from *Lablab purpureus *nodules in Papua New Guinea [11]. The complete genome of strain NGR234 has been sequenced [30]. Very recently, the classification of NGR234 was changed from *Rhizobium *sp to *Sinorhizobium fredii*. However, no genomic sequence of a type strain of *S. fredii *is available at present. Genome analysis of other *S. fredii *strains, both, typical and bean-nodulating, would help to define if the sequence migrated to a plasmid in a *S. fredii *ancestor, or in a more recent event.

The segment containing sequences similar to the *R. etli *transmissible plasmid pRet42a includes the genes involved in conjugative transfer. Conjugative transfer of *Agrobacterium tumefaciens *pTi and other rhizobial plasmids is subject to quorum-sensing regulation [3,4,31]. In pRet42a, transcription of *tra *and *trb *genes is activated by the autoinducer TraI and the transcriptional regulators TraR and CinR. The repressor encoded by *traM *is not active [5]. Plasmid pSfr64a contains similar regulatory genes, indicating that its transfer is probably regulated by quorum-sensing. Some differences, such as absence of *cinR *may account for specific responses to different host-related or environmental conditions. Preliminary data indicate the participation of new elements for the activation of the conjugative transfer of pSfr64a.

A comprehensive study of the regulatory mechanisms governing pSfr64a transfer will be addressed in the future.

We have shown that the pSym of GR64 is able to perform pSfr64a-dependent conjugative transfer. The process could be similar to what occurs in CFN42, where pRet42a forms a cointegrate with the pSym, allowing its transfer. Alternatively, pSfr64b mobilization could be induced *in trans*. The analysis of this process will be pursued in the future.

*R. etli *plasmid p42a was defined as self-transmissible because it may be transferred from diverse genomic backgrounds, such as *Agrobacterium*, containing no other plasmids [5,32]. The conjugation experiments performed in this work, show that pRet42a transfer is significantly decreased in GR64 background, suggesting the presence of host-specific elements that interfere with the transfer function. Regarding pSfr64a, conjugation occurs at high frequency when the donor is the native strain. Transfer has not been determined from plasmid-less strains, so that the lack of transfer from *R. etli *background could be due to the presence of an inhibitor, or to the lack of a required factor, encoded in the chromosome or pSfr64b. These data suggest that a plasmid may be "sequestered" by a host, and imply that the plasmid needs to adjust the appropriate expression of conjugal transfer functions to the new host environment.

## Conclusions

Bean-nodulating *S. fredii *strain GR64 carries a conjugative plasmid (pSfr64a) that has a large segment similar to the *R. etli *pSym, including replication, but not symbiosis-related genes, another segment similar to pRet42a, containing the transfer region, and a third segment, similar to the *S. fredii *NGR234 chromosome. The generation of this plasmid can be explained by the transfer of a symbiotic-conjugative-plasmid cointegrate from *R. etli *to a *S. fredii *strain; at least two recombination events among the *R. etli *plasmids and the *S. fredii *genome need to be invoked to explain the chimeric composition of plasmid pSfr64a. The structure of the symbiotic plasmid of GR64 could also be the result of these recombination events. Plasmid pSfr64a is required for conjugative transfer of the symbiotic plasmid. In spite of the similarity among pSfr64a and *R. etli *pRet42a conjugation related genes, the transfer process of these plasmids shows a host-specific behaviour.

## Methods

### Bacterial strains and plasmids

The bacterial strains and plasmids used in this work are described in Table 1. *R. etli *strains were grown at 30°C on PY medium [33]. *Escherichia coli *and *Agrobacterium tumefaciens *strains were grown on Luria-Bertani (LB) medium [34] at 37°C and 30°C respectively. When required, antibiotics were added at the following concentrations (in μg ml^-1^): nalidixic acid (Nal) 20, spectinomycin (Sp) 75, kanamycin (Km) 15, neomycin (Nm) 60, rifampicin (Rif) 100, streptomycin (Str) 50, tetracycline (Tc) 2 for *Rhizobium *and 10 for *E. coli*, gentamicin (Gm), and erythromycin (Ery) 100.

### Genetic manipulations

Conjugation experiments were performed on PY plates at 30°C, using overnight cultures grown to stationary phase. Donors and recipients were mixed in a 1:2 ratio and incubated overnight. The mixtures were collected and suspended in 1 ml of 10 mM MgSO_4_-0.01% Tween 40 (vol/vol). Serial dilutions were plated on suitable selective media. The transfer frequency was expressed as the number of transconjugants per donor.

A derivative of GR64 carrying a Tn*5mob*-labeled pSym was constructed by mating GR64 with strain S-17/pSUP5011 and selecting for resistance to Nal and Nm. Tagged plasmids were mobilized to *A. tumefaciens *GMI9023 [35] in triparental crosses, using pRK2013 [36] as helper, and selecting for Rif^R ^Nm^R ^transconjugants. Transconjugants carrying the tagged pSym (pSfr64b) were identified using Eckhardt type gels.

To determine the presence of transmissible plasmids, we randomly labeled strain GR64 with Tn*5*-GDYN, by mating it with *E. coli *S17/Tn*5*-GDYN [17] and selecting Nal^R ^Sp^R ^transconjugants. The labeled transconjugants were used as donors in conjugations with *A. tumefaciens *strain GMI9023. As the transposon integrates randomly into the chromosome or plasmids present in a strain, its integration into a transmissible plasmid confers a selective marker to the plasmid. Plasmids present in the selected transconjugants were visualized with Eckhardt gels.

The Tn5-GDYN element contains the *sacR-sacB *genes, which confer sucrose sensitivity in several gram-negative bacteria, so that selection of sucrose-resistant colonies allows the isolation of plasmid-less derivatives [17]. Plasmid-curing was carried out by plating overnight cultures of the transposon-labeled strains on PY plates containing 12.5% sucrose. Sucrose-resistant colonies were selected and verified as Sp^S^. Plasmid profiles of such colonies were analyzed in Eckhardt type gels.

### Construction of *S. fredii *and *R etli *derivatives with diverse plasmid content

We constructed various derivatives of GR64 (Table 1): GR64-1 has pSfr64a labeled with Tn*5*-GDYN and pSfr64b with Tn*5mob*. This construct allowed us to obtain a derivative cured of pSfr64a (GR64-2). The absence of pSfr64a in GR64-2 was confirmed by Southern type hybridization of plasmid profiles probed with purified pSfr64a (Figure 1B), and of total restricted DNA (data not shown). Tn*5*-GDYN-labeled-pRet42a from *R. etli *CFN42 was introduced into GR64-2 to generate GR64-3. A derivative of GR64-2 with a Tn*5*-GDYN inserted in pSfr64b was constructed. This strain was used to generate GR64-4, cured of both plasmids. Tn*5*-GDYN-labeled-pRet42a from *R. etli *CFN42 was introduced into GR64-4 to generate GR64-5. To construct GR64-6, Tn*5*-GDYN-labeled-pSfr64a was introduced into GR64-4. CFN2001 is a derivative of *R. etli *CFN42 that lacks the pSym (pRet42d), and the self-transmissible plasmid pRet42a [37]. This strain was used as receptor to select transconjugants carrying the Tn*5mob*-labeled pSym of GR64 (CFN2001-1), the Tn*5mob*-labeled pSym of GR64 and Tn*5*-GDYN-labeled pRet42a of *R. etli *CFN42 (CFN2001-2), and both plasmids of GR64 (CFN2001-3). Other derivatives carried either pSfr64a::Tn*5*-GDYN or pSfr64b::Tn*5mob *in *Agrobacterium *strain GMI9023 genomic background.

### Plasmid profiles

Plasmid profiles were visualized by the Eckhardt technique [38], as modified by Hynes and McGregor [39].

### Filter blot hybridization and plasmid visualization

For Southern-type hybridizations [40], Eckhardt type gels, or 1% agarose gels where restricted DNA was electrophoresed, were blotted onto nylon membranes, and hybridized under stringent conditions, as previously reported [41], by using Rapid-hyb buffer. Probes were linearized by digesting them with appropriate restriction enzymes and were labeled with [α^32^P]dCTP by using a Rediprime DNA labeling system. All restriction endonucleases, [α -^32^P]dCTP, hybridization buffer, and labeling systems were purchased from Amersham Pharmacia Biotech.

### Nodulation assays

Overnight cultures were used to inoculate surface-sterilized *Phaseolus vulgaris *cv. Negro Jamapa seeds. Plants were grown in 250-ml Erlenmeyer flasks with Fahraeus agar medium [42], without added nitrogen, at 28°C. Nodulation was scored at day 15 after inoculation. Surface-sterilized nodules were crushed on PY plates, and the plasmid pattern of single colonies was checked on Eckhardt type gels.

### Amplification and sequencing of *recA, rpoB*, and *nifH *gene fragments

Partial *nifH, recA *and *rpoB *fragments were amplified with the primer pairs *nifH*40F/*nifH*817R, recA41F/recA640R and rpoB454F/rpoB 1364R as previously described [43,44]. All amplifications were performed with *Taq *polymerase (USB-Amersham). Amplification products were purified using Roche's PCR product purification system. Both strands were commercially sequenced by Macrogen, Korea.

### Phylogenetic inference

Reference *nifH, recA, rpoB *and *repB *sequences were retrieved via BLASTP searches from a locally maintained BLAST database containing all fully sequenced Rhizobiales genomes, and via remote BLASTP searches against NCBI's non-redundant database. The query sequences for *nifH*, *recA *and *rpoB *used in the BLASTP searches were those obtained from the sequenced PCR amplicons from strain GR64, while that of *repB *was obtained from the sequence of pSfr64a. Nucleotide sequences were translated and aligned using muscle 3.7 [45]. The resulting protein multiple sequence alignments were used as masks to generate the underlying codon alignments using custom Perl scripts.

Models of nucleotide substitution were selected by the Akaike information criterion (AIC), using MODELTEST3.7 [46]. Among-site rate variation was modelled by a gamma distribution, approximated with 4 rate categories, each category being represented by its mean. Maximum likelihood (ML) trees were inferred under the AIC-selected models of nucleotide substitution for each data set using PhyML v3.0.1 [47]. The robustness of the ML topologies was evaluated using a recently developed Shimodaira-Hasegawa-like test for branches implemented in PhyML v3.0.1 [47]. For the sake of clarity, a small selection of the most relevant sequences was performed to show herein, based on the results of the phylogenetic analysis with the full set of homologous sequences.

### Sequencing of plasmid pSfr64a

Plasmid pSfr64a was purified by the Hirsch method [48], and used to construct a shotgun library with inserts of approximately 1-2 kb. A total of 1970 high-quality readings were collected by using the ABI3730XL automatic DNA sequencing machine (Applied Biosystems, Foster City, CA). Gaps were filled in by performing appropriate PCR amplification. Assemblages were obtained by the PhredPhrap-Consed software [49-51]. The quality of the final assembly was less than 1 error per 100,000 bases and had an average coverage of 6.5X.

### Annotation

Open reading frames were predicted by using GLIMMER 3.0 [52,53] and annotation was carried out with the help of BLASTX [54] comparisons against the GenBank nonredundant database [55], INTERPRO [56] searches, and manual curation by using ARTEMIS [57]. To compare partial genomic sequences with the nonredundant database of GenBank, BLASTX searches were performed, and the top hits were classified with respect to organisms with which they matched.

### Nucleotide sequence accesion number

Plasmid pSfr64a accession number is GenBank: CP002245. GR64 *nifH, recA*, and *rpoB *accesion numbers are respectively GenBank: JN034672, JN034673, JN034674.

## Competing interests

The authors declare that they have no competing interests.

## Authors' contributions

LC carried out most of the molecular genetics experiments. PB assembled the sequence, performed annotation and sequence alignments. LG participated in the design and performed some of the molecular genetics experiments. RIS obtained the sequence, and participated in the annotation and preparation of some illustrations. GD designed the sequencing strategy, participated in its analysis and prepared some of the illustrations. PV performed the phylogenetic analyses. DR participated in the design of the study and in the discussion of results. SB conceived the study, participated in its design and coordination and drafted the manuscript. All authors read and approved the final manuscript.

## Supplementary Material

Additional file 1 **Similarity of pSfr64a ORFs to genes located in the chromosome of NGR234, pRet42a and pRet42d plasmids**. Lists all the ORFs of pSfr64a, their predicted function, e-value and % of identity to the corresponding ORFs with highest similarity, located on the chromosome of *S. fredii *NGR234, and *R. etli *plasmids pRet42a and pRet42d. Click here for file
