# Supplementary material for: The conjugative plasmid of a bean-nodulating Sinorhizobium fredii strain is assembled from sequences of two Rhizobium plasmids and the chromosome of a Sinorhizobium strain
Source: BMC Microbiol. 2011 Jun 25;11:149. doi: 10.1186/1471-2180-11-149 (PMC3224233; doi:10.1186/1471-2180-11-149)
Supplement: Additional file 1 — Similarity of pSfr64a ORFs to genes located in the chromosome of NGR234, pRet42a and pRet42d plasmids. Lists all the ORFs of pSfr64a, their predicted function, e-value and % of identity to the corresponding ORFs with highest similarity, located on the chromosome of S. fredii NGR234, and R. etli plasmids pRet42a and pRet42d. [file 1471-2180-11-149-S1.PDF]

Additional file 1. Similarity of SFR64a ORFs to genes located in the chromosome of NGR234, pRet42a and pRet42d plasmids.

pSfr64a	NGR234	Retp42d	Retp42a	e-val (% of identity)	Predicted function
00001					hypothetical protein
00002					hypothetical conserved protein
**00003**					putative resolvase protein
00004					hypothetical conserved protein
00005					hypothetical conserved protein
00006					hypothetical conserved protein
00007					DNA mismatch endonuclease Vsr
00008					hypothetical conserved protein
**00009**					putative integrase catalytic protein
00010					IstB ATP binding domain-containing protein
00011					hypothetical protein
00012					putative ATPase AAA_5 protein
00013					dicarboxylate transporter, DctP subunit
00014					Dicarboxylate transport protein
00015	c06650			0.0 (81%)	methyl-accepting chemotaxis receptor
00016	c06120			1e-14 (62%)	type III effector outers protein
00017	c06130			0.0 (86%)	aldehyde dehydrogenase (ALDDH)
00018	c06140			e-120 (85%)	haloacid dehalogenase protein
00019	c06150			3e-31 (65%)	hypothetical conserved protein
00020	c06160			e-170 (79%)	3-isopropylmalate dehydrogenase
00021	c06170			2e-83 (77%)	transcriptional regulator, GntR family

00022	c06180			e-142 (82%)	UDP-glucose 4-epimerase protein
00023	c06190			e-108 (67%)	component of ABC transporter
00024	c06200			0.0 (79%)	ribose ABC transporter ATP-binding protein
00025	c06210			e-152 (74%)	ABC transporter sugar-binding protein
00026					hypotetical protein
00027	c06570			0.0 (86%)	transketolase I protein CbbT
00028	c06580			e-159 (81%)	fructose-1,6-bisphosphatase protein CbbF
00029	c06590			e-178 (89%)	fructose-bisphosphate aldolase protein CbbA2
00030	c06610			e-110 (75%)	triosephosphate isomerase protein TpiA
00031	c06620			2e-62 (78%)	sugar-phosphate isomerase protein
00032	c06380			2e-86 (68%)	transcriptional regulator protein
00033	c06410			2e-93 (69%)	3-oxoacyl-reductase FabG2
00034	c06420			8e-99 (45%)	hypothetical conserved protein
00035	c06430			4e-95 (60%)	hypothetical conserved protein
00036	c06440			0.0 (69%)	putative ABC transporter
00037	c06530			1e-81 (56%)	putative ABC transporter permease protein
00038	c06520			e-143 (80%)	solute-binding component of ABC transporter
00039	c06510			e-118 (71%)	aldo/keto reductase protein
00040	c06470			e-148 (63%)	ribulose-bisphosphate carboxylase RbcL
00041	c06560			e-113 (61%)	putative oxidoreductase protein

00042					Sua5/YciO/YrdC/YwlC family protein
00043	c06500			e-162 (81%)	putative aldo/keto reductase protein
00044	c06480			e-132 (77%)	putative beta-lactamase protein
00045	c06600			5e-61 (49%)	transcriptional regulator protein, DeoR family
00046		PD00036*[1]		e-118 (95%)	5-amino-6-uracil reductase
00047		PD00037		e-105 (69%)	probable oxidoreductase protein
00048					hypothetical protein
00049		PD00038		e-140 (97%)	oxidoreductase protein
00050		PD00039		e-117 (92%)	oxidoreductase protein
00051		PD00040*[2]		e-160 (94%)	transcriptional regulator protein
00052	c06240			0.0 (78%)	aminoacyl-histidine dipeptidase protein PepD
00053	c06250			8e-71 (81%)	hypothetical conserved protein
00054	c06260			4e-60 (80%)	hypothetical conserved protein
00055	c06270			e-169 (82%)	putative ribose ABC transporter protein
00056	c06280			0.0 (76%)	ribose ABC transporter ATP-binding protein
00057	c06290			e-122 (72%)	ribose ABC transporter protein
00058	c06300			e-125 (99%)	L-ribulose-5-phosphate 4-epimerase protein
00059	c06310			0.0 (84%)	ribulokinase protein
00060	c06320			e-152 (87%)	transcriptional regulator protein, LysR family
00061		PD00082		1e-91 (77%)	transcriptional regulator protein, GntR family
00062		PD00083		9e-97 (66%)	6-phosphogluconate dehydrogenase

					protein
00063		PD00084		4e-61 (81%)	decarboxylase
00064		PD00085		1e-54 (80%)	hypothetical conserved protein
00065		PD00086		e-166 (93%)	putative carboxylesterase protein
00066		PD00087		0.0 (92%)	ribose ABC transporter
00067		PD00088		0.0 (78%)	ribose ABC transporter, ATP-binding protein
00068		PD00089		e-134 (78%)	ribose ABC transporter, permease protein
00069		PD00090		e-116 (66%)	ribose ABC transporter, permease protein
00070		PD00091*[3]		0.0 (85%)	succinate-semialdehyde dehdyrogenase GabD
00071					hypothetical protein
00072		PD00126*[4]		e-170 (79%)	aryl-alcohol dehydrogenase protein
00073		PD00128		0.0 (87%)	periplasmic sugar binding protein TeuB
00074		PD00129		0.0 (81%)	sugar transport ATP-binding protein TeuA
00075		PD00130		e-132 (77%)	sugar transport system permease TeuC1
00076		PD00131		e-130 (74%)	sugar transport system permease TeuC2
00077		PD00132		0.0 (88%)	aryl-alcohol dehydrogenase protein
00078		PD00133		3e-30 (63%)	hypothetical conserved protein
00079		PD00134		0.0 (84%)	NADH dehydrogenase protein
00080					hypothetical protein

00081		PD00102*[5]		2e-81 (66%)	oxidoreductase protein
00082		PD00103		0.0 (68%)	methyl-accepting chemotaxis protein McpA
00083		PD00104		0.0 (79%)	putative sugar ABC transporter protein
**00084**					putative transposase protein
00085		PD00137		0.0 (96%)	probable two-component sensor
00086		PD00136		0.0 (86%)	methyl-accepting chemotaxis protein McpC
00087		PD00135		e-166 (93%)	putative transcriptional regulator AraC family
**00088**					probable transposase protein
00089		PD00121*[6]		5e-82 (72%)	GTP cyclohydrolase II protein
00090		PD00120		1e-34 (50%)	NAD(P)H-dependent FMN reductase
00091		PD00090		7e-80 (50%)	ribose ABC transporter, permease protein
00092		PD00089		9e-77 (56%)	ribose ABC transporter, permease protein
00093		PD00088		0.0 (69%)	ribose ABC transporter, ATP-binding protein
00094					hypothetical protein
00095		PD00119		5e-57 (82%)	putative formaldehyde-activating protein
00096		PD00118		0.0 (79%)	succinyldiaminopimelate aminotransferase
00097		PD00117		0.0 (82%)	sugar ABC transporter
00098		PD00116		0.0 (91%)	monooxygenase protein
00099		PD00115		2e-59 (82%)	gamma-carboxymuconolactone

					decarboxylase
00100		PD00114		4e-66 (74%)	4-hydroxyphenylacetate-3-monooxygenase
00101		PD00113		e-105 (76%)	short chain dehydrogenase protein
00102		PD00110		e-155 (64%)	putative hydrolase protein
00103		PD00082		1e-87 (73%)	probable transcriptional regulator protein
00104		PD00108		4e-64 (67%)	putative beta-lactamase protein
00105		PD00107		e-151 (70%)	alcohol dehydrogenase protein
00106		PD00106		e-131 (75%)	putative metallo-hydrolase protein
00107					methyl-accepting chemotaxis protein MclA
00108		PD00043*[7]		0.0 (89%)	beta-glucosidase protein
00109					hypothetical conserved protein
00110		PD00106		3e-90 (55%)	putative beta-lactamase protein
00111					hypothetical protein
00112					hypothetical protein
00113					C4-dicarboxylate transporter, DctQ and DctM
00114					TRAP dicarboxylate transporter, DctP subunit
00115					6-phosphogluconate dehydrogenase protein
00116					alpha/beta hydrolase fold related protein
00117					transcriptional regulator, LysR family
00118					xylose isomerase-like TIM barrel

					protein
00119					glycerone kinase protein
00120					dihydroxyacetone kinase, L subunit
00121					hypothetical conserved protein
00122			PA00139¥	e-179 (91%)	hypothetical conserved protein
00123			PA00140	e-105 (93%)	hypothetical conserved protein
00124			PA00141	0.0 (89%)	DNA methylase protein
00125			PA00142	3e-18 (97%)	hypothetical conserved protein
00126			PA00143	0.0 (89%)	ParB-like plasmid partitioning protein
00127			PA00144	4e-72 (85%)	hypothetical conserved protein
00128			PA00145	e-147 (86%)	hypothetical conserved protein
00129			PA00146	2e-40 (75%)	hypothetical conserved protein
00130			PA00147	5e-65 (88%)	hypothetical conserved protein
00131			PA00148	4e-82 (82%)	hypothetical conserved protein
00132			PA00149	e-155 (88%)	antirestriction protein ArdC
00133			PA00150	6e-49 (86%)	hypothetical conserved protein
00134			PA00151	3e-53 (100%)	hypothetical conserved protein
00135			PA00152	5e-29 (60%)	hypothetical conserved protein
00136			PA00153	1e-70 (83%)	hypothetical conserved protein
00137			PA00153.1†	2e-70 (83%)	hypothetical conserved protein
00138			PA00154	1e-28 (92%)	hypothetical conserved protein
00139			PA00155	2e-81 (78%)	putative nuclease protein
00140			PA00156	0.0 (78%)	conjugation protein TraG
00141			PA00157	3e-16 (66%)	conjugation protein TraD
00142			PA00158	2e-23 (93%)	conjugation protein TraC

00143			PA00159	0.0 (88%)	conjugation protein TraA
00144			PA00160	4e-82 (81%)	conjugation protein TraF
00145			PA00161	e-177 (77%)	conjugation protein TraB
00146			PA00162	1e-82 (75%)	conjugation protein TraH
00147					hypothetical conserved protein
00148					hypothetical conserved protein
00149			PA00165¶	6e-05 (32%)	XRE family transcriptional regulator
00150			PA00166¶	3e-21 (55%)	conjugation repressor protein TraM
00151			PA00167¶	2e-69 (53%)	transcriptional activator protein TraR
00152			PA00169	0.0 (80%)	conjugation protein TrbI
00153			PA00170	2e-40 (71%)	conjugation protein TrbH
00154			PA00171	e-135 (89%)	conjugation protein TrbG
00155			PA00172	e-117 (95%)	conjugation protein TrbF
00156			PA00173	e-131 (67%)	conjugation protein TrbL
00157			*trbK* §	8e-11 (70%)	conjugation protein TrbK
00158			PA00174	e-112 (89%)	conjugation protein TrbJ
00159			PA00175	0.0 (92%)	conjugation protein TrbE
00160			PA00176	8e-47 (92%)	conjugation protein TrbD
00161			PA00177	6e-36 (63%)	conjugation protein TrbC
00162			PA00178	e-157 (87%)	conjugation protein TrbB
00163			PA00179¶	2e-79 (70%)	autoinducer synthesis protein TraI
00164		PD00352		0.0 (97%)	plasmid partitioning protein RepAa
00165		PD00353		e-152 (89%)	plasmid partitioning protein RepBa

00166		PD00354		e-177 (79%)	plasmid replication protein RepCa

SFR64A transposon-related ORFSs are marked in bold.

* Indicates that there is a nearby ORF on pRet42d, encoding a transposon-related protein: PD00033*[1], PD00041*[2], PD00093*[3], PD00124*[4], PD00101*[5], PD00123*[6], PD00041*[7]

¥ Indicates that the adjacent ORF on pRet42a (PA00138) encodes a transposon-related protein

† New ORF identified in pRet42a, by tblastn with the product of 00137

¶ Conserved localization in both plasmids, although highest similarity is not to pRetCFN42a, but to other *R.etli* replicons.

§ New ORF identified by comparison with *Rhizobium etli* CFN42 plasmid p42a transfer region, complete sequence (NCBI accesion. AF528525.1)
